# Early B cell transcriptomic markers of measles-specific humoral immunity following a 3^rd^ dose of MMR vaccine

**DOI:** 10.3389/fimmu.2024.1358477

**Published:** 2024-04-03

**Authors:** Iana H. Haralambieva, Jun Chen, Huy Quang Quach, Tamar Ratishvili, Nathaniel D. Warner, Inna G. Ovsyannikova, Gregory A. Poland, Richard B. Kennedy

**Affiliations:** ^1^ Mayo Clinic Vaccine Research Group, Department of Internal Medicine, Mayo Clinic, Rochester, MN, United States; ^2^ Department of Quantitative Health Sciences, Mayo Clinic, Rochester, MN, United States

**Keywords:** MMR vaccine, measles vaccine, measles virus, humoral immunity, gene expression, B cells

## Abstract

B cell transcriptomic signatures hold promise for the early prediction of vaccine-induced humoral immunity and vaccine protective efficacy. We performed a longitudinal study in 232 healthy adult participants before/after a 3^rd^ dose of MMR (MMR3) vaccine. We assessed baseline and early transcriptional patterns in purified B cells and their association with measles-specific humoral immunity after MMR vaccination using two analytical methods (“per gene” linear models and joint analysis). Our study identified distinct early transcriptional signatures/genes following MMR3 that were associated with measles-specific neutralizing antibody titer and/or binding antibody titer. The most significant genes included: the interleukin 20 receptor subunit beta/*IL20RB* gene (a subunit receptor for IL-24, a cytokine involved in the germinal center B cell maturation/response); the phorbol-12-myristate-13-acetate-induced protein 1/*PMAIP1*, the brain expressed X-linked 2/*BEX2* gene and the B cell Fas apoptotic inhibitory molecule/*FAIM*, involved in the selection of high-affinity B cell clones and apoptosis/regulation of apoptosis; as well as *IL16* (encoding the B lymphocyte-derived IL-16 ligand of CD4), involved in the crosstalk between B cells, dendritic cells and helper T cells. Significantly enriched pathways included B cell signaling, apoptosis/regulation of apoptosis, metabolic pathways, cell cycle-related pathways, and pathways associated with viral infections, among others. In conclusion, our study identified genes/pathways linked to antigen-induced B cell proliferation, differentiation, apoptosis, and clonal selection, that are associated with, and impact measles virus-specific humoral immunity after MMR vaccination.

## 1 Introduction

Omics and systems biology studies in vaccinology investigate how immune parameters are perturbed after vaccination at the whole systems level, and endeavor to identify transcriptomic/omics markers and models that can serve as immune response “signatures” correlated with or predictive of outcomes such as vaccine immunogenicity and/or protective efficacy (1–5). Most of these studies focus on humoral immune responses, as they are crucial for protection against many viral pathogens. Humoral immunity is conferred by antibodies (Ab) and the B lymphocytes/plasma cells that produce them, with the important contribution of CD4^+^ T cell help (6). Both the initial plasmablast response and the generated pools of long-lived plasma cells and memory B cells have significant role in protection, in maintaining Ab responses, and in carrying out the anamnestic response upon subsequent viral exposure.

Measles virus (MV) is part of the live attenuated MMR vaccine containing measles, mumps, and rubella, which has been effective in reducing the morbidity and mortality associated with these three pathogens, although with differing degrees of success (1, 2). A third dose of MMR vaccine (MMR3) is administered in outbreak settings to control mumps, and more rarely during measles outbreaks (7). Here in this study, we used MMR vaccine as a probe and a model system to study transcriptomic signatures of the recall B cell response in individuals known to be high and low antibody responders to the measles component of the vaccine. We comprehensively investigated early transcriptional events in purified B cells of 232 study participants and their impact/association with humoral immunity after MMR vaccination. Our results demonstrate distinct transcriptional patterns after receipt of MMR3, which are correlated with, and may explain the observed inter-individual differences in, measles vaccine-induced humoral immunity.

## 2 Materials and methods

**Figure 1:**
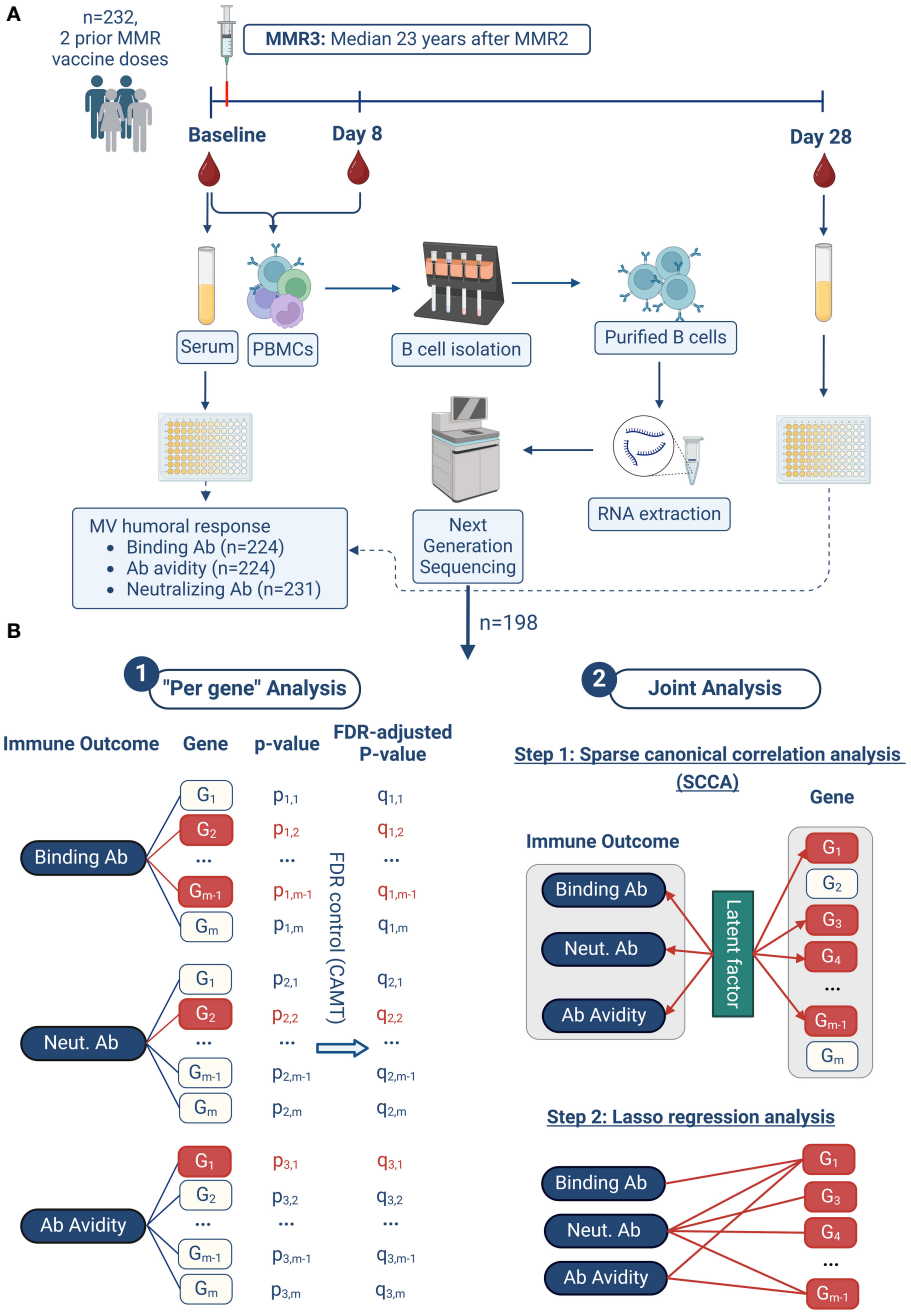
Study design and analysis approach. The workflow of our study is illustrated in **(A)**. Our two-pronged analysis approach in summarized in **(B)** and consists of two major steps: 1. “per gene” analysis, and 2. joint analysis. 1. In the "Per gene" analysis model, a linear regression model is fitted for each immune outcome and each gene, with the immune outcome as the dependent variable and the gene expression as the independent variable, adjusting for other covariates. Group-adaptive false discovery rate control (FDR) using the CAMT procedure is then performed based on these individual association P-values. Genes with FDR-adjusted P-values less than 0.1 are considered significant (highlighted in red). 2. In the joint analysis model, the three immune outcomes and all gene expressions are analyzed together, promoting the selection of genes associated with multiple humoral immune outcomes. This analysis proceeds in two steps. Firstly, sparse canonical correlation analysis (SCCA) is applied to select a subset of genes (highlighted in red) whose expressions are most correlated with the three immune outcomes through latent factors. Secondly, lasso sparse regression is applied for each immune outcome based on the SCCA-selected genes from the previous step. The result of this second step is a detailed association network between the three immune outcomes and the associated genes. This figure was created with BioRender.com.

The described methods are similar or identical to the ones in our previously published studies (8–13). Our study design/workflow and analysis methodology are outlined in **Figure 1**.

### 2.1 Study participants

The study cohort has been previously described in detail (11, 13). It is comprised of 232 healthy subjects from Olmsted County (MN, USA) with two prior documented doses of MMR vaccine. Study subjects provided blood samples prior to the receipt of MMR3 vaccine (Day 0, baseline) and at Day 8 and Day 28 following vaccination. Demographic and clinical variables were collected, including age, sex, race, ethnicity, and MMR vaccination history, as described in our previous study (13). The study was approved by The Mayo Clinic Institutional Review Board. All enrolled participants for the study provided written informed consent.

### 2.2 Measles virus-specific binding antibody and avidity

MV-specific IgG antibody titer was measured using the Zeus ELISA Measles IgG Test System (Zeus Scientific, Inc., Branchburg, NJ), and results are presented as sample index (SI), as previously described (13). Per the kit’s instructions, a sample index greater than 1.1 indicates a seropositive sample. The assay had an intra-assay coefficient of variability (CV) of 6.7% and inter-assay CV of 7.2% in our laboratory.

MV-specific IgG avidity was measured using the Zeus ELISA Measles IgG Test System as previously described (13). Avidity was calculated as the percentage of the absorbance value with and without diethylamine (DEA) in washing buffer. Low avidity (below 30%) and moderate/high avidity (above 30%) were defined arbitrarily using a previously established avidity threshold (11).

### 2.3 Measles virus-specific neutralizing antibody

Neutralizing antibodies were measured using an optimized MV Edmonston-specific fluorescence-based plaque reduction microneutralization assay, as previously described (8, 10). The 50% neutralizing dose (ND_50_) was calculated using Karber’s formula, and the ND_50_ titer was converted to mIU/mL using the 3^rd^ anti-measles serum international standard (NIBSC code No. 97/648) (8, 10). The assay had a CV of 5.7% and a limit of detection of 15 mIU/mL in our laboratory.

### 2.4 mRNA sequencing

Next-generation mRNA sequencing was performed in purified B cells as previously described (12). B cells were first isolated from PBMCs via negative selection using the Miltenyi Biotec’s B cell isolation kit and MidiMACS™ Separator. This process yielded B cells with an average cell viability (measured by Trypan blue exclusion test) of 98% and average B cell purity (assessed by flow cytometry) of 93%. Total RNA was extracted from the isolated bulk B cells using the RNeasy Plus Mini Kit (Qiagen, Valencia, CA), and evaluated for quality/concentration on an Agilent 2010 Bioanalyzer (Agilent, Palo Alto, CA).

cDNA libraries were generated at the Mayo Clinic’s Gene Sequencing Core according to the manufacturer’s protocol using the TruSeq^®^ Stranded mRNA Library Prep v2 kit (Illumina, San Diego, CA). Illumina’s NovaSeq 6000 S2 Reagent Kit (100 cycles) was used to perform paired-end read sequencing on the Illumina NovaSeq 6000 Instrument. The MAP-RSeq version 3.0 pipeline was applied to align reads using STAR to the hg38 human reference genome, and gene expression counts were obtained using featureCounts utilizing the gene definition files from Ensembl v78 (14). Conditional Quantile Regression was used for normalization (15).

### 2.5 Statistical analysis

Genes with low abundance or less variability were filtered out (median count <16 at each timepoint or <20^th^ percentile of CV), and a total of 10,174 genes were included in the analyses. The analysis was performed separately for the Baseline and Day 8 gene expression data. The immune outcome was defined as the difference between Day 28 immune outcome and baseline (i.e., Day 28 – Day 0 difference/change on the linear scale). The immune outcomes assessed included: change in MV-specific binding Ab (anti-MV IgG) presented as sample index (SI), change in MV-specific IgG avidity calculated as the percentage/ratio of the ELISA absorbance value with and without the chaotropic agent/DEA (avidity index/AI) and change in anti-MV nAb in mIU/mL (Neut. Ab mIU/ml), as previously described (13).

Our analysis approach is summarized in **Figure 1B**. It consisted of two major steps. In the first step, we focused on the “per gene” model since this statistical approach is standard and commonly applied. The advantage of this approach is the maturity of the statistical method (linear regression), the explicit error control (false discovery rate control) and the ability to retrieve correlated genes (thus facilitating enrichment analysis), while the disadvantage is reduction of statistical power (discussed below and in Results). In the second step of our analysis, we applied the joint analysis approach, which addresses some of the limitations of the “per gene” analysis and applies a selection of a sparse subset of genes, which jointly have the highest correlation with the overall vaccine-induced immunity (the three humoral immune outcomes).

“Per gene” model was fitted using multiple linear regression with each immune outcome as the response, and the gene expression as the predictor, controlling for batch, age, and gender effects. Linear model-based t-test was used to calculate “per gene” p-values, followed by multiple testing correction using group-adaptive false discovery rate (FDR) control based on the Covariate Adaptive Multiple Testing/CAMT procedure (16, 17). Here, the group structure is specified by the immune outcome the p-values come from. FDR-corrected p-value or q-value less than 0.1 was used as the significance cutoff. Enriched gene pathways were identified using the Gene Set Enrichment Analysis method (GSEA, (18)), as implemented in the “gseKEGG” (GSEA of KEGG) function of the R Bioconductor package “clusterProfiler” v4.6.2 (19). In comparison to the over-representation test based on the significant genes only, GSEA examines the ranks of the effect sizes (e.g., log2 fold change) for all genes in a specific pathway, and if the rank is overall higher or lower than what would be expected from a random distribution, it indicates that the pathway is activated or suppressed. In our gene expression dataset, Entrez Gene IDs were available for 9,479 of the 10,174 analyzed genes, which were then used in the GSEA. Gene coefficient estimates from the “per gene” models were used as the effect size. FDR control (Benjamini-Hochberg procedure) was performed based on the enrichment p-values (20) to correct for multiple testing.

To complement the “per gene” modeling results, we performed joint analysis of all genes and the three immune outcomes together with the goal to reveal additional biological insights into the influence of gene expression on vaccine-induced immune response outcomes (21). Since the same gene could simultaneously be associated with multiple immune outcomes, joint analysis of all the three immune outcomes could increase the statistical power to identify such co-associated genes. To do this, we first used sparse canonical correlation analysis (SCCA) (22), which selects a sparse subset of genes that explains the most correlation between the gene expression data from a specific timepoint (baseline or Day 8) and the three humoral immune outcomes (using the R “PMA” package v1.2-2). Permutation test was used to select the sparsity tuning parameter as implemented in the “cca.permute” function of the R “PMA” package. Since SCCA does not associate the genes to a specific humoral immune outcome, we further proceeded to identify the genes associated with each of the specific humoral immune outcomes. We applied the least absolute shrinkage and selection operator/lasso regression model to the SCCA-selected genes (all selected genes or the top 500 genes based on the largest SCCA coefficients, if more than 500 genes were selected) for each humoral immune outcome (R “glmnet” package 4.1-8) (23). To account for covariates, linear regression was used to control for confounding variables (effects of batch, gender and age), and the residuals were used in the SCCA. Cross-validation was used to select the sparsity tuning parameter as implemented in the “cv.glmnet” function of the R “glmnet” package. All the statistical analyses were performed in R 4.1.2.

## 3 Results

### 3.1 Characteristics of the study cohort and humoral immune response outcomes after MMR3

The study cohort has been previously described in detail and is comprised of two subcohorts as previously described (13). The demographic characteristics of our study cohort was reflective of the demographics of the Olmsted County, MN population (U.S.). According to their racial characteristics the study participants were mostly White (96.5%), and their ethnicity was mostly non-Hispanics or Latino (95.3%). The study cohort included 62.9% females, the median age at enrollment was 35.95 years (IQR 31.95, 40.9) and the study participants’ median body mass index (BMI) was 27.9. Median ages at the first dose and second dose of MMR were 15.59 months (IQR 15, 17.71), and 12.5 years (IQR 11.43, 17.15), respectively. In 1998, the American Academy of Pediatrics recommended the current MMR vaccine schedule (2^nd^ dose at 4-6 years of age). A significant portion of our cohort was older than 4-6 years of age at the time of these recommendations and therefore received the ‘catch-up’ dose (second dose of MMR vaccine) upon entering their next school (middle school/junior high or high school). In the course of this study participants received a third MMR vaccine dose approximately 23 years (median 23.45 years) after their second MMR vaccine dose (**Figure 1**). The immune outcomes for the study cohort are summarized in **Supplementary Figure 1**. All humoral immune outcomes increased significantly from baseline to Day 28 following MMR3 vaccination (*p* < 2.3E^-08^ for all immune outcomes), indicating a significant boost of measles-specific humoral immunity, as previously described (13). At baseline the median nAb titer for the study cohort was 535 mIU/mL (IQR: 260, 1250), and at the peak (Day 28) of antibody response after MMR3, the median of nAb titer was 845 mIU/mL (IQR: 421, 1694). The median Day 28 sample index was 3.47 (IQR: 2.55, 4.21) and the median Ab avidity was 42.8% (IQR: 33.74, 55.43), as previously described (13). Importantly, considerable variation in each humoral immune outcome was observed in our study cohort (13) – providing an ideal scenario for evaluating the potential role of MMR3-induced transcriptional changes in B cells in association with such immune response variability.

Of the study cohort, 198 participants had gene expression data on Day 0 and Day 8 (see **Figure 1**), as well as neutralizing antibody measure (Day 0 and Day 28) and were used in the transcriptomic association analysis (with neutralizing Ab). Of the subjects with gene expression data, MV-specific binding Ab (SI) and Avidity measures were available for 191 subjects at Day 0 and for 194 subjects at Day 28, and therefore the transcriptomic association analysis with these immune measures was performed in 191 subjects. The Day 0 (baseline) and Day 8 gene expression patterns (heatmaps) across covariates (sex, age, subcohort) and MV-specific immune response outcomes (neutralizing Ab, binding Ab and avidity) are displayed in **Supplementary Figures 2**, **3**.

### 3.2 Baseline B cell transcriptomic markers associated with MV-specific humoral immune response following MMR vaccination ("per gene" linear models)

For our analyses, the humoral immune response to vaccination (MV-specific binding IgG Ab, IgG avidity, and neutralizing Ab) was defined as the difference/change of Day 28 immune outcome with respect to baseline (i.e., Day 28 – Day 0 defined as a difference).

#### 3.2.1 Results of “per gene” linear model analysis reveal the impact of B cell Day 0/baseline gene expression on MV-specific humoral immunity after MMR vaccination

**Figure 2:**
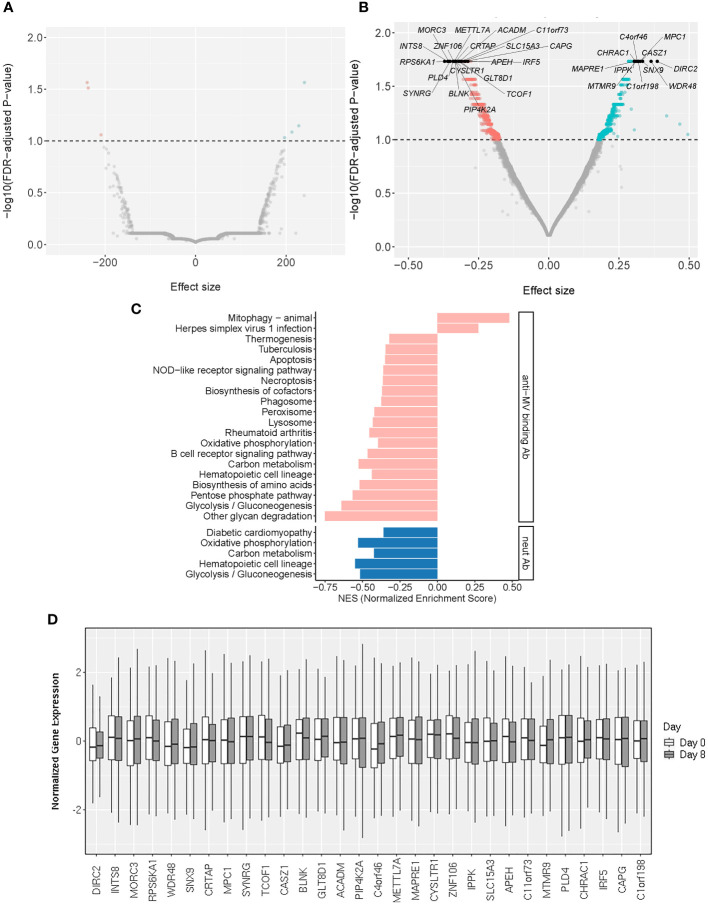
Baseline transcriptomic markers associated with MV-specific humoral immunity. **(A, B)** The volcano plots illustrate the association of baseline (Day 0) gene expression with neutralizing Ab **(A)** and MV-specific binding Ab **(B)**. The effect size represents the coefficient from the “per gene” linear regression analysis. The top 30 significant genes are designated with their gene symbols. **(C)** Pathway enrichment analysis/GSEA plots of hallmark pathways for Day 0 gene associations with MV-specific humoral immunity (binding Ab/pink or neutralizing Ab/blue). NES was calculated based on the coefficients from “per gene” analysis. **(D)** Normalized gene expression box plots of the top (most significant) 30 Day 0 (baseline) genes associated with MV-specific humoral immunity across two timepoints (Day 0 and Day 8).

First, we assessed the “per gene” associations between baseline/Day 0 gene expression and the Day 28 – Day 0 humoral immune response outcomes. The “per gene” linear model was fitted for each humoral immune response outcome separately. We identified 1,152 B-cell genes displaying significant associations (q-value < 0.1) with measures of MV-specific vaccine-induced humoral immunity, although their individual effect (see Coefficient, **Table 1**) on the immune response was relatively small (**Table 1**; **Figures 2A, B**). Of the most statistically significant genes, several (e.g., B cell linker/BLNK, interferon regulatory factor 5/IRF5, phosphatidylinositol-5-phosphate 4-kinase type 2 alpha/PIP4K2A, all with q-value = 0.0185) are known to impact various B cell activities and functions.

**Table 1:** “Per gene” linear model analysis for Day 0 gene expression in B cells and Day 28 – Day 0 humoral immune response outcomes.

Gene Symbol	Description	Outcome	p-value	q-value	Coefficient*
* DIRC2*	solute carrier family 49 member 4	SI- ELISA binding Ab	3.25E-07	0.0185	0.386
* INTS8*	integrator complex subunit 8	SI- ELISA binding Ab	2.75E-06	0.0185	-0.361
* MORC3*	MORC family CW-type zinc finger 3	SI- ELISA binding Ab	3.52E-06	0.0185	-0.357
* RPS6KA1*	ribosomal protein S6 kinase A1	SI- ELISA binding Ab	3.60E-06	0.0185	-0.372
* WDR48*	WD repeat domain 48	SI- ELISA binding Ab	4.92E-06	0.0185	0.364
* SNX9*	sorting nexin 9	SI- ELISA binding Ab	8.64E-06	0.0185	0.334
* CRTAP*	cartilage associated protein	SI- ELISA binding Ab	8.94E-06	0.0185	-0.331
* MPC1*	mitochondrial pyruvate carrier 1	SI- ELISA binding Ab	1.04E-05	0.0185	0.324
* SYNRG*	synergin gamma	SI- ELISA binding Ab	1.14E-05	0.0185	-0.352
* TCOF1*	treacle ribosome biogenesis factor 1	SI- ELISA binding Ab	1.90E-05	0.0185	-0.322
* CASZ1*	castor zinc finger 1	SI- ELISA binding Ab	2.34E-05	0.0185	0.326
* BLNK*	B cell linker	SI- ELISA binding Ab	2.40E-05	0.0185	-0.334
* GLT8D1*	glycosyltransferase 8 domain containing 1	SI- ELISA binding Ab	3.07E-05	0.0185	-0.316
* ACADM*	acyl-CoA dehydrogenase medium chain	SI- ELISA binding Ab	3.15E-05	0.0185	-0.318
* PIP4K2A*	phosphatidylinositol-5-phosphate 4-kinase type 2 alpha	SI- ELISA binding Ab	3.55E-05	0.0185	-0.333
* C4orf46*	chromosome 4 open reading frame 46	SI- ELISA binding Ab	3.76E-05	0.0185	0.315
* METTL7A*	methyltransferase like 7A	SI- ELISA binding Ab	3.76E-05	0.0185	-0.336
* MAPRE1*	microtubule associated protein RP/EB family member 1	SI- ELISA binding Ab	3.92E-05	0.0185	0.304
* CYSLTR1*	cysteinyl leukotriene receptor 1	SI- ELISA binding Ab	3.94E-05	0.0185	-0.333
* ZNF106*	zinc finger protein 106	SI- ELISA binding Ab	4.21E-05	0.0185	-0.339
* IPPK*	inositol-pentakisphosphate 2-kinase	SI- ELISA binding Ab	5.16E-05	0.0185	0.305
* SLC15A3*	solute carrier family 15 member 3	SI- ELISA binding Ab	6.03E-05	0.0185	-0.301
* APEH*	acylaminoacyl-peptide hydrolase	SI- ELISA binding Ab	6.33E-05	0.0185	-0.313
* C11orf73*	heat shock protein nuclear import factor hikeshi	SI- ELISA binding Ab	6.99E-05	0.0185	-0.310
* MTMR9*	myotubularin related protein 9	SI- ELISA binding Ab	7.16E-05	0.0185	0.309
* PLD4*	phospholipase D family member 4	SI- ELISA binding Ab	7.51E-05	0.0185	-0.341
* CHRAC1*	chromatin accessibility complex subunit 1	SI- ELISA binding Ab	7.73E-05	0.0185	0.309
* IRF5*	interferon regulatory factor 5	SI- ELISA binding Ab	7.92E-05	0.0185	-0.288
* CAPG*	capping actin protein, gelsolin like	SI- ELISA binding Ab	8.09E-05	0.0185	-0.298
* C1orf198*	chromosome 1 open reading frame 198	SI- ELISA binding Ab	8.48E-05	0.0185	0.319

The top 30 displayed genes/findings with significant associations are genes associated with SI/anti-MV IgG as an immune outcome (see Statistical analysis).

*Coefficient can be interpreted as the change of the immune outcome measurement in response to one standard deviation change of the gene expression.

#### 3.2.2 Pathway enrichment analysis on Day 0 gene expression

From a systems biology point of view, pathways and even seemingly unrelated “pools” of different genes may be collectively important. To untangle the biological processes behind our “per gene” models we performed pathway enrichment analysis on the Day 0 genes associated with anti-MV binding or neutralizing antibody titer after MMR vaccination, as described in Statistical analysis. Our assessment confirmed the enrichment of genes involved in metabolic pathways and basic cellular/organelle functions (lysosome, phagosome), as well as signal transduction pathway genes linked to inflammation/autoimmunity (e.g., NOD-like receptor signaling pathway) and/or host innate and adaptive immune response, including the B cell receptor signaling pathway (**Figures 2C, D**; **Supplementary Table 1**).

### 3.3 Early/Day 8 B-cell transcriptomic markers associated with MV-specific humoral immune response following MMR vaccination (“per gene” linear models)

#### 3.3.1 Results of “per gene” linear model analysis reveal the impact of early B cell gene expression on MV-specific humoral immunity after MMR vaccination

**Figure 3:**
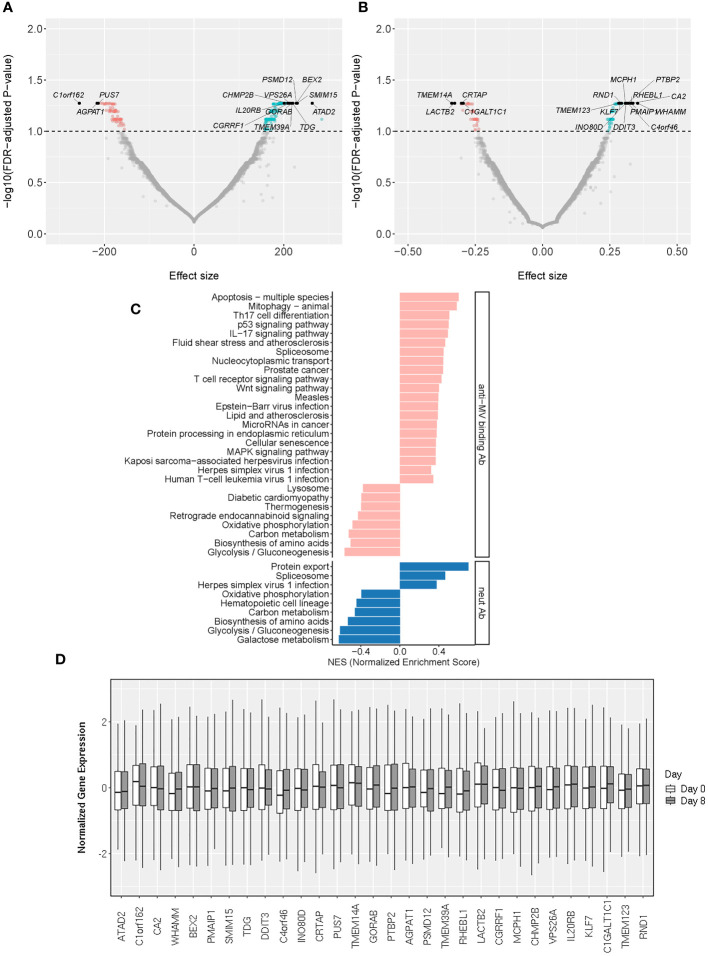
Early/Day 8 transcriptomic markers associated with MV-specific humoral immunity. **(A, B)** The volcano plots illustrate the association of Day 8 gene expression with neutralizing Ab **(A)** and MV-specific binding Ab **(B)**. The effect size represents the coefficient from the “per gene” linear regression analysis. The top 30 significant genes are designated with their gene symbols. **(C)** Pathway enrichment analysis/GSEA plots of hallmark pathways for Day 8 gene associations with MV-specific humoral immunity (binding Ab/pink or neutralizing Ab/blue). NES was calculated based on the coefficients from “per gene” analysis. **(D)** Normalized gene expression box plots of the top (most significant) 30 Day 8 genes associated with MV-specific humoral immunity across two timepoints (Day 0 and Day 8).

The early transcriptional events in B cells upon antigenic stimulation are of critical importance for the generation and maintenance of humoral immunity. Since other studies have reported associations between plasmablast transcriptional response (peaking at Day 7-8) and antibody titers following vaccination, our study sought to identify early (Day 8, plasmablast) transcriptional signatures in B cells that are highly correlated with vaccine-induced humoral immune outcomes (24–27). To achieve this, we fit linear models for each gene with the Day 8 gene expression as the covariate. While this “per gene” analysis yielded a smaller number of significantly associated genes (n=318, Day 8 genes with statistically significant associations at q-value < 0.1), compared to baseline genes, their individual gene effects/weights (reported as an estimated effect of each gene/Coefficient, **Table 2**; **Figures 3A, B**) on the immune response outcome were relatively large, which is consistent with the substantial contribution of specific Day 8 B-cell genes to the measured immune outcome. Of note, among the top 30 most significant findings we identified interleukin 20 receptor subunit beta/*IL20RB*, phorbol-12-myristate-13-acetate-induced protein 1/*PMAIP1* and brain expressed X-linked 2/*BEX2* gene involved in apoptosis, proteasome 26S subunit, non-ATPase 12/*PSMD12*, involved in ubiquitination and replication of influenza virus, and other genes linked to antigen-induced proliferation, differentiation, apoptosis, commitment to different B cell lineages and clonal selection (**Table 2**; **Figure 3**).

**Table 2:** “Per gene” linear model results for Day 8 B-cell gene expression and Day 28 – Day 0 humoral immune outcomes.

Gene Symbol	Description	Outcome	p-value	q-value	*Coefficient
* ATAD2*	ATPase family AAA domain containing 2	Neut. Ab miu/ml	1.51E-06	0.053	263.35
* C1orf162*	chromosome 1 open reading frame 162	Neut. Ab miu/ml	3.23E-06	0.053	-255.78
* CA2*	carbonic anhydrase 2	SI ELISA binding Ab	5.71E-06	0.053	0.34
* WHAMM*	WASP homolog associated with actin, golgi membranes and microtubules	SI ELISA binding Ab	8.44E-06	0.053	0.35
* BEX2*	brain expressed X-linked 2	Neut. Ab miu/ml	1.26E-05	0.053	228.33
* PMAIP1*	phorbol-12-myristate-13-acetate-induced protein 1	SI ELISA binding Ab	1.44E-05	0.053	0.33
* SMIM15*	small integral membrane protein 15	Neut. Ab miu/ml	2.37E-05	0.053	229.99
* TDG*	thymine DNA glycosylase	Neut. Ab miu/ml	2.56E-05	0.053	219.91
* DDIT3*	DNA damage inducible transcript 3	SI ELISA binding Ab	2.91E-05	0.053	0.31
* C4orf46*	chromosome 4 open reading frame 46	SI ELISA binding Ab	3.26E-05	0.053	0.32
* INO80D*	INO80 complex subunit D	SI ELISA binding Ab	3.48E-05	0.053	0.31
* CRTAP*	cartilage associated protein	SI ELISA binding Ab	3.69E-05	0.053	-0.30
* PUS7*	pseudouridine synthase 7	Neut. Ab miu/ml	4.47E-05	0.053	-213.79
* TMEM14A*	transmembrane protein 14A	SI ELISA binding Ab	5.57E-05	0.053	-0.34
* GORAB*	golgin, RAB6 interacting	Neut. Ab miu/ml	6.10E-05	0.053	218.02
* PTBP2*	polypyrimidine tract binding protein 2	SI ELISA binding Ab	6.11E-05	0.053	0.32
* AGPAT1*	1-acylglycerol-3-phosphate O-acyltransferase 1	Neut. Ab miu/ml	6.58E-05	0.053	-216.29
* PSMD12*	proteasome 26S subunit, non-ATPase 12	Neut. Ab miu/ml	7.07E-05	0.053	227.86
* TMEM39A*	transmembrane protein 39A	Neut. Ab miu/ml	7.23E-05	0.053	218.20
* RHEBL1*	RHEB like 1	SI ELISA binding Ab	7.51E-05	0.053	0.33
* LACTB2*	lactamase beta 2	SI ELISA binding Ab	7.88E-05	0.053	-0.33
* CGRRF1*	cell growth regulator with ring finger domain 1	Neut. Ab miu/ml	8.26E-05	0.053	211.89
* MCPH1*	microcephalin 1	SI ELISA binding Ab	8.57E-05	0.053	0.31
* CHMP2B*	charged multivesicular body protein 2B	Neut. Ab miu/ml	8.71E-05	0.053	208.85
* VPS26A*	VPS26, retromer complex component A	Neut. Ab miu/ml	8.78E-05	0.053	213.23
* IL20RB*	interleukin 20 receptor subunit beta	Neut. Ab miu/ml	9.84E-05	0.053	201.08
* KLF7*	Kruppel like factor 7	SI ELISA binding Ab	9.99E-05	0.053	0.29
* C1GALT1C1*	C1GALT1 specific chaperone 1	SI ELISA binding Ab	0.0001	0.053	-0.30
* TMEM123*	transmembrane protein 123	SI ELISA binding Ab	0.0001	0.053	0.28
* RND1*	Rho family GTPase 1	SI ELISA binding Ab	0.0001	0.053	0.29

Top 30 genes/findings included associations with SI/anti-MV IgG and neutralizing Ab (see Statistical analysis).

*Coefficient can be interpreted as the change of the immune outcome measurement in response to one standard deviation change of the gene expression.

#### 3.3.2 Pathway enrichment analysis on Day 8 gene expression

This assessment identified 29 significantly enriched pathways (q<0.05) among the genes associated with MV-specific binding antibody and 9 significantly enriched pathways among the genes associated with MV-specific nAb. We observed a moderate overlap with the enriched Day 0 gene expression pathways/**Figure 3C**; **Supplementary Table 2**) consisting of basic metabolic and cellular function-related pathways. Among the identified enriched pathways, there were also five pathways associated with different viral infections (measles virus, herpes simplex virus, Kaposi sarcoma-associated herpesvirus, human T cell leukemia virus 1 and Epstein-Barr virus) and multiple pathways related to metabolism, basic cellular functions, signaling pathways and lymphocyte immune activity (**Figure 3C**; **Supplementary Table 2**).

### 3.4 Results from joint analysis of B-cell transcriptomic markers associated with MV-specific humoral immune response following MMR vaccination

“Per gene” model tests one gene at a time and requires multiple testing correction that may result in reduced statistical power. In addition, the “per gene” model aggregates the effects of other relevant genes into the error term, thus increasing the variance of the error term and reducing the statistical power to identify genes with moderate effects. If a gene is associated with multiple immune outcomes, the “per gene” model is not able to use such information, leading to further loss of statistical power. “Per gene” model also does not account for correlations among genes, and highly correlated genes tend to be selected together. Thus, it has limited ability to identify genes, whose associations are independent of other genes. Joint analysis of gene expression, on the other hand, could address some of the listed limitations and reveal additional biological insights. To achieve this, we performed a sparse canonical correlation analysis (SCCA) to first select genes jointly impacting the three MV-specific humoral immune outcomes (neutralizing Ab, binding Ab/SI and avidity/AI). Since the same gene could simultaneously be associated with multiple immune outcomes, joint analysis of all the three immune outcomes could increase the statistical power to identify these co-associated genes. We then performed lasso regression analysis on the SCCA-selected genes to identify genes associated with a specific humoral immune outcome (see Statistical analysis).

#### 3.4.1 Results from joint gene expression analysis of the impact of baseline B-cell gene expression on MV-specific humoral immunity after vaccination

**Figure 4:**
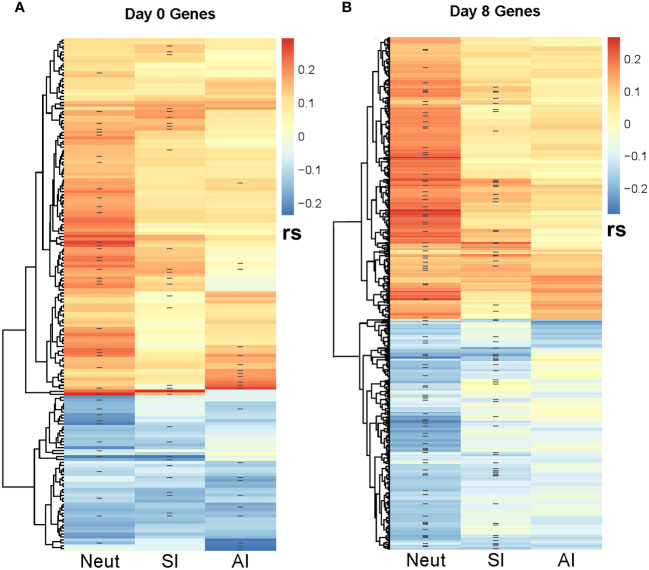
Correlation heatmap between SCCA-selected genes and MV-specific humoral immune response outcomes. The heatmaps illustrate the Spearman correlations (rs) between the SCCA-selected genes and the three immune response outcomes (neutralizing Ab/NeutAb, binding antibody sample index/SI and avidity index/AI). ‘-’ denotes the genes selected by the Lasso regression analysis as associated with each immune outcome. **(A)** illustrates the Spearman correlations with immune outcomes for the Day 0 (baseline) genes. **(B)** illustrates the Spearman correlations with immune outcomes for the Day 8 genes. As shown, many genes are co-associated with multiple immune outcomes.

The SCCA analysis on the baseline B cell gene expression resulted in the selection of 172 genes simultaneously associated with all three measures of MV-specific humoral immunity (nAb, binding Ab, and antibody avidity **Figure 4A**). The lasso regression analysis of these genes resulted in the identification of 40 genes associated with MV-specific neutralizing antibody, 31 genes associated with MV-specific binding antibody and 22 genes associated with antibody avidity, including predominantly metabolic genes and genes involved in different cell signaling cascades (**Supplementary Table 3**).

#### 3.4.2 Results from joint gene expression analysis of the impact of early/Day 8 B-cell gene expression on MV-specific humoral immunity after vaccination

The SCCA assessment on the Day 8 B-cell gene expression led to the selection of many genes associated with the three measures of MV-specific humoral immunity (n=7,716). Although it is possible that a large number of genes are associated with the activation of B cells, each with weak effects, we recognize that the large number of genes selected could also be due to the limitation of the lasso sparsity penalty used in SCCA, where it tends to produce a denser model in order to retain those truly associated genes. Thus, we focused our further analysis on the top 500 genes with largest SCCA coefficients (**Figure 4B**). Focusing on the top 500 selected genes, the lasso regression analysis identified 94 genes associated with MV-specific neutralizing antibody and 66 genes associated with MV-specific binding antibody (**Supplementary Table 4**). Of note, although the analytical approaches we used were different, we observed a reasonable number of overlapping results with the identified genes via “per gene” linear models. For example, of the 94 genes associated with MV-specific nAb (identified via the joint analysis approach), 6 genes (*PUS7, TDG, PTBP2, BEX2, CRTAP, INO80D*) were among the 30 top genes (representing 20% of the top genes associated with MV-specific humoral immunity) that were identified via “per gene” linear models (see **Table 2**). Thirty one of the 94 genes (approximately 33%) overlapped with the list of the 318 significantly associated genes (q <0.1) with humoral immunity via “per gene” linear models. Among these overlapping genes, most had an unknown link to B cells and/or the generation/maintenance of humoral immunity, however a few were known apoptotic genes (e.g., BEX2, involved in the regulation of mitochondrial apoptosis, as well as the Fas apoptotic inhibitory molecule/FAIM, **Supplementary Table 4**) that may have implications on the process of apoptosis in the B cell lineage. Finally, the joint analysis approach identified also many non-shared (with the results from the linear models) genes, among those the interleukin 16/*IL16* gene associated with the nAb titer (**Supplementary Table 4**).

## 4 Discussion

The discovery of genes/genetic signatures or other “omics” measurements associated with and/or predictive of immune response after vaccination has been the goal and the subject of cutting-edge systems-level vaccine research for over a decade (3–5, 28).

The current study identified multiple key biomarkers/factors and pathways that contribute to and shape inherent B cell activity and functions necessary for generating and/or maintaining optimal vaccine-induced humoral immunity. We focused our study design on the B cell compartment in order to identify intrinsic B cell factors driving the recall immune response to vaccination and highly associated with MV-specific humoral immunity. We acknowledge that our study design (i.e., measuring gene expression in purified B cells) supported the identification of B cell-specific genes, even so this study identified a range of distinct early transcriptional activities (transcriptional factors) and specific molecular/cellular processes, that influence recall measles-specific humoral immunity. One of our most important findings is the discovery of *IL20RB* gene (encoding a cytokine receptor subunit of the heterodimeric complex required for IL-19, IL-20 and IL-24 binding and activity) as an early transcriptional biomarker in B cells that was highly associated with MV-specific nAb titer. The interleukin/IL-20 subfamily consists of IL-19, IL-20, IL-22, IL-24 and IL-26, and its members are involved in inflammatory and innate immune (including antiviral) activity, tissue repair/homeostasis, cell communication, proliferation and differentiation, and oncogenesis (29). Of the known cytokines using this receptor/subunit, IL-24 has been described as a pivotal B cell immunoregulatory cytokine, directly involved in the processes of germinal center B cell maturation (30). This multifunctional cytokine signals through two heterodimeric receptors IL-20RA/IL-20RB and IL-20RB/IL22RA1 (both include the subunit encoded by *IL20RB*) and is known to mediate inflammatory and autoimmune responses, as well as to regulate a variety of immune cell functions (including in B cells, T cells, NK cells, and macrophages) (29). Although not specifically linked to vaccine-induced immunity, IL20RB and the associated signaling pathway have been identified as critical in the protection and host defense against mucosal pathogens (31). It has been postulated that BCR activation/CD40 engagement (CD40-CD40L ligation) in follicular B cells (in particular CD27^+^ memory B cells and CD5^+^ B cells) is associated with high expression of IL-24, which plays an important role in supporting germinal center T-dependent antigen B cell proliferation (30). The ligand IL-24 has been shown to hinder plasma cell/terminal B cell differentiation and antibody production by favoring the maturation of memory B cells (30). Interestingly, we previously identified IL-24 (*IL24*) along with CD93 as markers of differential MV-specific transcriptional response (in PBMCs) in 15 high *vs*. 15 low antibody responders to measles vaccination (32). Hence, it is likely that the differential expression of IL-24 receptor and/or IL-24 by specific B cell subsets during B cell activation (early post-measles vaccine), physiologically “fine-tunes” the balance between plasma cell and MBC commitment, thus affecting antigen-specific plasmablast/plasma cell response and antibody production. We speculate that further investigation in this direction can potentially lead to the development of improved vaccine candidates by modulating the production of IL-24 via: incorporating an adjuvant that stimulates IL-24 production; incorporating a recombinant IL-24 lacking apoptosis-inducing properties (33); or generating a recombinant virus, expressing IL-24 or a factor silencing IL-24 for testing in future studies. Another interesting early B cell transcriptional marker associated with antibody response is the phorbol-12-myristate-13-acetate-induced protein 1/*PMAIP1* (Noxa), encoding a pro-apoptotic member of the BCL-2 protein family with significant involvement in the selection of high-affinity B cell clones upon antigenic stimulation (34, 35). The ablation of the encoded protein leads to increased survival of low-affinity clones at the expense of high-affinity clones *in vivo*, in a mouse model following influenza vaccination (34–36).

The two analytical approaches (linear models and joint analysis) used in our study have discovered that approximately 20-30% of the identified genes overlap and are associated with immune outcome/nAb titer, which builds confidence in our findings. Both approaches identified genes involved in the apoptosis/regulation of apoptosis. For example, the *BEX2* gene is a known regulator of mitochondrial apoptosis and G1 cell cycle, while *FAIM* (cloned as an inhibitor/regulator of Fas-mediated apoptosis in B cells) has a significant role in the regulation of germinal center B cell response and the plasma cell compartment response (37–39). Another important gene, *IL16* (encoding the B lymphocyte-derived IL-16 ligand of CD4), identified via the joint analysis approach, has been demonstrated to play a significant role in the crosstalk and attraction/recruitment of dendritic cells and helper T cells to initiate and achieve an optimal humoral immune response (40, 41). A vaccine study in solid organ transplant patients, found that IL-16 levels (among other cytokines) were significantly lower in subjects with very low antibody response to mRNA-based COVID-19 vaccine compared to subjects with normal immune response, suggesting that this cytokine is associated with the optimal development of humoral immunity after COVID-19 vaccination (42).

Another highlighted finding in our study has been identified as a novel virus-specific host factor. The proteasome 26S subunit, non-ATPase 12/*PSMD12*, has been previously implicated in regulation of the replication/budding of influenza virus through K63-specific ubiquitination of the matrix/M1 viral structural protein (43). It is plausible that it may impact the budding/replication of other enveloped RNA viruses, and thus affect antigenic abundance/host response. Factors associated with anti-vital immunity, such as IRF5 (identified in our study) were found to be part of a molecular signature induced by LAIV influenza vaccination (44, 45). In agreement with the identified genes/cellular functions, our pathway enrichment analysis of Day 0 and Day 8 genes/gene expression pointed to enriched pathways associated with different viral infections, as well as to multiple cytokines and immune/B cell signaling pathways, apoptosis/regulation of apoptosis, metabolic pathways and cell cycle-related pathways, among others. As expected, we found pathways and gene expression patterns that have been previously identified with other viral vaccines and immune response studies. A member of the B cell signaling pathway triggered upon B cell activation (TNFRSF17, a receptor for BLyS-BAFF) was identified as a key predictive factor for neutralizing antibody response to yellow fever vaccination (24). B cell signaling modules were also identified as important for the optimal response to influenza vaccination (46). Other identified pathways in our study (apoptosis/regulation of apoptosis) have also been found to impact immune response to vaccination by others. Furman et al., identified the regulation of apoptosis as an essential pathway prognostic of responsiveness to influenza vaccine (47). Vaccine adjuvants and vaccine components, conversely, were demonstrated to induce damage-associated molecular patterns (DAMPs) and cell death-associated signaling pathways, that were found to be important for augmenting immunogenicity after vaccination (48, 49). As separate studies found associations between genes regulating apoptosis and immune response to vaccination, it is likely that apoptosis and its regulation may play a role (perhaps through increased survival of antibody producing cells) in vaccine-induced immunity. This warrants further investigation.

The strengths of our study include the relatively large (for transcriptomic studies) sample size, the acquisition of high-quality transcriptomic information/data from purified B cells before/after MMR vaccination and the use of two different analytical approaches to identify biologically relevant gene signatures. An important limitation is the possibility of false positive findings, which is alleviated with the reporting of FDR-adjusted p-values or q-values and the implementation of the joint analysis. Using the FDR control, the percentage of false positives is controlled in the “per gene” analysis. While the joint analysis method does not offer explicit FDR control, by jointly analyzing the immune outcomes and the genes together, the method promotes the selection of genes associated with multiple outcomes, thus pooling association evidence across immune outcomes. The two steps of our analysis (“per gene” model and joint analysis) are complementary rather than competitive. Together, the results they produce provide a better understanding of the important transcriptional factors underlying measles vaccine-induced humoral immunity. Another important point to mention is the confounding effect of simultaneous immune stimulation during MMR vaccination (measles, mumps, and rubella). In this regard, it will be important to study the effect of the identified genes on rubella virus and mumps virus-specific immune outcomes. It is also important to note, that although our goal was to study Day 8 (plasmablast) transcriptional response in terms of association with humoral immunity, the assessment of earlier transcriptional programs in B cells (collected at earlier timepoints) could provide additional valuable insights into the generation of recall immune response after vaccination. Validation of our major findings through functional studies is necessary to determine the contribution of specific gene/genes (e.g., *IL20RB*) to MV-specific humoral immunity. Another avenue to explore is the assessment of transcriptional patterns (including the identified genes of high interest and other genes) in different B cell subsets at different timepoints following vaccination, which will help to better understand the gene expression dynamics in the B cell compartment and its contribution to humoral immunity.

In summary, our study identified important early B lymphocyte-derived transcriptomic signatures (*IL20RB*, *PMAIP1*, *BEX2*, *FAIM*, and *IL16*) associated with functional immunity/MV-specific neutralizing antibody response and other measures of humoral immunity following MMR vaccination. We suggest that such molecular signatures can serve as early biomarkers of optimal vaccine immunogenicity and hold promise for (potentially) improving vaccine-induced immunity through providing useful information for the development of next-generation vaccine candidates (3, 46, 50, 51).

## Data availability statement

The data presented in this study are deposited in Synapse.org (Sage Bionetworks) under Project SynID syn54153421, at https://www.synapse.org/#!Synapse:syn54153421/wiki/626686. The publicly available dataset consists of subjects who consented to data sharing.

## Ethics statement

The studies involving humans were approved by The Mayo Clinic Institutional Review Board. The studies were conducted in accordance with the local legislation and institutional requirements. The participants provided their written informed consent to participate in this study.

## Author contributions

IH: Data curation, Investigation, Methodology, Writing – original draft. JC: Formal analysis, Software, Writing – review & editing. HQ: Data curation, Investigation, Methodology, Writing – review & editing. TR: Investigation, Methodology, Writing – review & editing. NW: Formal analysis, Software, Writing – review & editing. IO: Investigation, Project administration, Supervision, Writing – review & editing. GP: Conceptualization, Funding acquisition, Writing – review & editing. RK: Conceptualization, Funding acquisition, Supervision, Writing – review & editing.

## Supplementary material

Supplementary Figure 1 Immune response summary of the study subjects. Box plots summarizing: **(A)** Day 0 and Day 28 binding antibody sample index/SI; **(B)** Day 0 and Day 28 antibody avidity index; and **(C)** Day 0 and Day 28 Neutralizing Ab. The line indicates the median of the immune response measure in our cohort, while the whiskers indicate 25% and 75% IQR. The p-values (Wilcoxon signed rank test) demostrate the significant upregulation of Day 28 immune outcomes (post MMR3) compared to baseline (Day 0) immune outcomes.

Supplementary Figure 2 Heatmap of Day 0 gene expression patterns. Heatmap of Day 0 (baseline) gene expression patterns of the significant genes (FDR < 0.1) from “per gene” analysis across covariates (sex, age, subcohort) and MV-specific immune response outcomes (Day 28 – Day 0 difference): Neutralizing Ab (Naut.Ab),SI (Sample Index/Binding Ab) and AI (Avidity Index).

Supplementary Figure 3 Heatmap of Day 8 gene expression patterns. Heatmap of Day 8 gene expression patterns of the significant genes (FDR < 0.1) from “per gene” analysis across covariates (sex, age, subcohort) and MV-specific immune response outcomes (Day 28 – Day 0 difference): Neutralizing Ab (Naut.Ab),SI (Sample Index/Binding Ab) and AI (Avidity Index).
