# Supplementary material for: Early B cell transcriptomic markers of measles-specific humoral immunity following a 3rd dose of MMR vaccine
Source: Front Immunol. 2024 Apr 3;15:1358477. doi: 10.3389/fimmu.2024.1358477 (PMC11021587; doi:10.3389/fimmu.2024.1358477)
Supplement: Supplementary Figure 1 — Immune response summary of the study subjects. Box plots summarizing: (A) Day 0 and Day 28 binding antibody sample index/SI; (B) Day 0 and Day 28 antibody avidity index; and (C) Day 0 and Day 28 Neutralizing Ab. The line indicates the median of the immune response measure in our cohort, while the whiskers indicate 25% and 75% IQR. The p-values (Wilcoxon signed rank test) demostrate the significant upregulation of Day 28 immune outcomes (post MMR3) compared to baseline (Day 0) immune outcomes. [file DataSheet_1.docx]

**Supplementary Table 1 Pathway enrichment analysis for the Day 0 B cell genes/gene expression associations with Day 28 – Day 0 immune outcome (anti-MV binding Ab and nAb)**

| **Pathway ID** | **Description** | **p-value** | **q-value** |
| --- | --- | --- | --- |
|  | **Enriched pathways of genes assoc.with anti-MV binding Ab** |  |  |
| hsa01200 | Carbon metabolism | 2.73E-07 | 7.79E-05 |
| hsa00010 | Glycolysis / Gluconeogenesis | 6.24E-07 | 8.91E-05 |
| hsa04142 | Lysosome | 9.87E-06 | 0.0009 |
| hsa00511 | Other glycan degradation | 0.0001 | 0.0099 |
| hsa01230 | Biosynthesis of amino acids | 0.0002 | 0.0103 |
| hsa05168 | Herpes simplex virus 1 infection | 0.0002 | 0.0108 |
| hsa01240 | Biosynthesis of cofactors | 0.0004 | 0.0159 |
| hsa04640 | Hematopoietic cell lineage | 0.0004 | 0.0159 |
| hsa05323 | Rheumatoid arthritis | 0.0005 | 0.0168 |
| hsa04621 | NOD-like receptor signaling pathway | 0.0006 | 0.0177 |
| hsa04137 | Mitophagy - animal | 0.0007 | 0.0190 |
| hsa00190 | Oxidative phosphorylation | 0.0009 | 0.0226 |
| hsa04146 | Peroxisome | 0.0013 | 0.0288 |
| hsa04662 | B cell receptor signaling pathway | 0.0017 | 0.0354 |
| hsa04217 | Necroptosis | 0.0024 | 0.0410 |
| hsa00030 | Pentose phosphate pathway | 0.0024 | 0.0410 |
| hsa04145 | Phagosome | 0.0024 | 0.0410 |
| hsa04714 | Thermogenesis | 0.0026 | 0.0412 |
| hsa05152 | Tuberculosis | 0.0028 | 0.0412 |
| hsa04210 | Apoptosis | 0.0029 | 0.0412 |
|  | **Enriched pathways of genes assoc.with neut Ab** |  |  |
| hsa00190 | Oxidative phosphorylation | 2.66E-08 | 8.53E-06 |
| hsa04640 | Hematopoietic cell lineage | 1.71E-07 | 2.75E-05 |
| hsa01200 | Carbon metabolism | 9.27E-05 | 0.0099 |
| hsa00010 | Glycolysis / Gluconeogenesis | 0.0003 | 0.0272 |
| hsa05415 | Diabetic cardiomyopathy | 0.0005 | 0.0299 |

* Enriched pathways with q-value below 0.05 are included

**Supplementary Table 2. Pathway enrichment analysis for the Day 8 B cell genes/gene expression associations with Day 28 – Day 0 humoral immune outcomes (anti-MV binding Ab and nAb)**

| **Pathway ID** | **Description** | **p-value** | **q-value** |
| --- | --- | --- | --- |
|  | **Enriched pathways of genes assoc.with anti-MV binding Ab** |  |  |
| hsa05168 | Herpes simplex virus 1 infection | 1.95E-07 | 5.38E-05 |
| hsa01200 | Carbon metabolism | 5.29E-07 | 7.29E-05 |
| hsa04137 | Mitophagy - animal | 3.93E-06 | 0.0003 |
| hsa00190 | Oxidative phosphorylation | 4.54E-06 | 0.0003 |
| hsa04714 | Thermogenesis | 1.73E-05 | 0.0009 |
| hsa04659 | Th17 cell differentiation | 1.93E-05 | 0.0009 |
| hsa00010 | Glycolysis / Gluconeogenesis | 4.69E-05 | 0.0018 |
| hsa05418 | Fluid shear stress and atherosclerosis | 6.10E-05 | 0.0021 |
| hsa04115 | p53 signaling pathway | 8.46E-05 | 0.0026 |
| hsa04010 | MAPK signaling pathway | 0.0001 | 0.0029 |
| hsa05415 | Diabetic cardiomyopathy | 0.0001 | 0.0029 |
| hsa05169 | Epstein-Barr virus infection | 0.0002 | 0.0035 |
| hsa05417 | Lipid and atherosclerosis | 0.0002 | 0.0035 |
| hsa04723 | Retrograde endocannabinoid signaling | 0.0002 | 0.0047 |
| hsa01230 | Biosynthesis of amino acids | 0.0003 | 0.0060 |
| hsa03040 | Spliceosome | 0.0005 | 0.0080 |
| hsa04142 | Lysosome | 0.0005 | 0.0080 |
| hsa05162 | Measles | 0.0011 | 0.0165 |
| hsa04657 | IL-17 signaling pathway | 0.0011 | 0.0165 |
| hsa05206 | MicroRNAs in cancer | 0.0014 | 0.0199 |
| hsa04141 | Protein processing in endoplasmic reticulum | 0.0016 | 0.0206 |
| hsa05167 | Kaposi sarcoma-associated herpesvirus infection | 0.0018 | 0.0229 |
| hsa04310 | Wnt signaling pathway | 0.0019 | 0.0229 |
| hsa04215 | Apoptosis - multiple species | 0.0025 | 0.0283 |
| hsa04660 | T cell receptor signaling pathway | 0.0034 | 0.0380 |
| hsa03013 | Nucleocytoplasmic transport | 0.0038 | 0.0385 |
| hsa05215 | Prostate cancer | 0.0039 | 0.0385 |
| hsa05166 | Human T-cell leukemia virus 1 infection | 0.0039 | 0.0385 |
| hsa04218 | Cellular senescence | 0.0041 | 0.0390 |
|  | **Enriched pathways of genes assoc.with neut Ab** |  |  |
| hsa05168 | Herpes simplex virus 1 infection | 1.00E-10 | 3.11E-08 |
| hsa00010 | Glycolysis / Gluconeogenesis | 2.75E-06 | 0.0004 |
| hsa01200 | Carbon metabolism | 3.16E-05 | 0.0033 |
| hsa01230 | Biosynthesis of amino acids | 9.33E-05 | 0.0072 |
| hsa03040 | Spliceosome | 0.0004 | 0.0218 |
| hsa04640 | Hematopoietic cell lineage | 0.0005 | 0.0253 |
| hsa00052 | Galactose metabolism | 0.0007 | 0.0300 |
| hsa03060 | Protein export | 0.0011 | 0.0414 |
| hsa00190 | Oxidative phosphorylation | 0.0013 | 0.0435 |

***** Enriched pathways with q< 0.05 are included.

**Supplementary Table 3** Results from SCCA and lasso regression modeling results of baseline B cell transcriptomic markers associated with MV-specific humoral immune response following MMR vaccination

| **Gene Symbol** | **Description** | **Beta Coefficient** | **Spear. p-value** | **Spearman**  **Correlation**  **Coefficient** |
| --- | --- | --- | --- | --- |
|  | Associations with Neut. Ab miu/ml |  |  |  |
| *BTN1A1* | butyrophilin subfamily 1 member A1 | 0.145 | 0.00003 | 0.292 |
| *PUS7* | pseudouridine synthase 7 | -0.133 | 0.066 | -0.131 |
| *WASL* | WASP like actin nucleation promoting factor | 0.114 | 0.023 | 0.161 |
| *SLC39A1* | solute carrier family 39 member 1 | 0.101 | 0.006 | 0.193 |
| *SLC39A14* | solute carrier family 39 member 14 | 0.093 | 0.001 | 0.227 |
| *STAU2* | staufen double-stranded RNA binding protein 2 | -0.085 | 0.032 | -0.152 |
| *RP11-1012A1.4* | novel protein | 0.083 | 0.011 | 0.180 |
| *RNASE4* | novel protein, ANG-RNASE4 readthrough | 0.072 | 0.009 | 0.184 |
| *LRRC16A* | capping protein regulator and myosin 1 linker 1 | 0.065 | 0.012 | 0.177 |
| *TTC39C* | tetratricopeptide repeat domain 39C | -0.060 | 0.002 | -0.222 |
| *FKBP5* | FKBP prolyl isomerase 5 | 0.057 | 0.0004 | 0.248 |
| *WDR60* | dynein 2 intermediate chain 1 | 0.056 | 0.014 | 0.174 |
| *MOB3B* | MOB kinase activator 3B | -0.054 | 0.078 | -0.125 |
| *LRRC69* | leucine rich repeat containing 69 | 0.050 | 0.003 | 0.207 |
| *PAK4* | p21 (RAC1) activated kinase 4 | 0.047 | 0.019 | 0.167 |
| *GPR89A* | G protein-coupled receptor 89A | -0.046 | 0.030 | -0.154 |
| *PI16* | peptidase inhibitor 16 | -0.045 | 0.008 | -0.189 |
| *E2F1* | E2F transcription factor 1 | 0.045 | 0.0001 | 0.268 |
| *CTC-360G5.8* | novel protein | -0.043 | 0.129 | -0.108 |
| *FAM133DP* | family with sequence similarity 133 member D, pseudogene | 0.041 | 0.064 | 0.132 |
| *API5P1* | apoptosis inhibitor 5 pseudogene 1 | 0.040 | 0.003 | 0.209 |
| *DYRK2* | dual specificity tyrosine phosphorylation regulated kinase 2 | -0.037 | 0.004 | -0.202 |
| *BMP8B* | bone morphogenetic protein 8b | 0.036 | 0.001 | 0.226 |
| *PGRMC2* | progesterone receptor membrane component 2 | 0.030 | 0.005 | 0.198 |
| *ROM1* | retinal outer segment membrane protein 1 | 0.028 | 0.018 | 0.168 |
| *TMEM184B* | transmembrane protein 184B | 0.024 | 0.011 | 0.181 |
| *CAMSAP2* | calmodulin regulated spectrin associated protein family member 2 | 0.022 | 0.002 | 0.219 |
| *USP31* | ubiquitin specific peptidase 31 | 0.016 | 0.021 | 0.164 |
| *ANP32E* | acidic nuclear phosphoprotein 32 family member E | 0.014 | 0.010 | 0.183 |
| *ATP6V1E2* | ATPase H+ transporting V1 subunit E2 | -0.013 | 0.002 | -0.222 |
| *ZNF222* | zinc finger protein 222 | 0.013 | 0.021 | 0.164 |
| *SULT1A3* | sulfotransferase family 1A member 3 | 0.012 | 0.008 | 0.189 |
| *BAI1* | adhesion G protein-coupled receptor B1 | 0.011 | 0.035 | 0.150 |
| *RP11-204C16.4* | YWHAZ pseudogene 10 | 0.010 | 0.008 | 0.187 |
| *MAPRE3* | microtubule associated protein RP/EB family member 3 | 0.010 | 0.001 | 0.228 |
| *DPM2* | dolichyl-phosphate mannosyltransferase subunit 2, regulatory | -0.009 | 0.141 | -0.105 |
| *RP11-395L14.17* | SNRPA1 pseudogene 1 | 0.005 | 0.058 | 0.135 |
| *SPINT1* | serine peptidase inhibitor, Kunitz type 1 | -0.004 | 0.269 | -0.079 |
| *AURKA* | aurora kinase A | 0.001 | 0.020 | 0.165 |
| *SLC30A4* | solute carrier family 30 member 4 | 0.000 | 0.015 | -0.172 |
|  | Associations with ELISA binding Ab/SI |  |  |  |
| *EXTL2* | exostosin like glycosyltransferase 2 | 0.124 | 0.00004 | 0.288 |
| *PUS7* | pseudouridine synthase 7 | -0.110 | 0.072 | -0.128 |
| *ROM1* | retinal outer segment membrane protein 1 | 0.092 | 0.007 | 0.192 |
| *USP31* | ubiquitin specific peptidase 31 | 0.082 | 0.016 | 0.172 |
| *CXorf57* | RPA1 related single stranded DNA binding protein, X-linked | 0.076 | 0.017 | 0.169 |
| *WASL* | WASP like actin nucleation promoting factor | 0.075 | 0.041 | 0.145 |
| *MOB3B* | MOB kinase activator 3B | -0.074 | 0.081 | -0.124 |
| *CTTNBP2NL* | CTTNBP2 N-terminal like | 0.067 | 0.012 | 0.178 |
| *RNASE4* | novel protein, ANG-RNASE4 readthrough | 0.063 | 0.052 | 0.138 |
| *SARS2* | seryl-tRNA synthetase 2, mitochondrial | -0.053 | 0.023 | -0.161 |
| *BTN1A1* | butyrophilin subfamily 1 member A1 | 0.052 | 0.040 | 0.146 |
| *ATP6V1E2* | ATPase H+ transporting V1 subunit E2 | -0.052 | 0.001 | -0.230 |
| *C7orf31* | chromosome 7 open reading frame 31 | -0.051 | 0.031 | -0.154 |
| *SETSIP* | SET like protein | 0.045 | 0.053 | 0.138 |
| *RMDN2* | regulator of microtubule dynamics 2 | -0.043 | 0.008 | -0.187 |
| *MGAT5* | alpha-1,6-mannosylglycoprotein 6-beta-N-acetylglucosaminyltransferase | 0.043 | 0.133 | 0.107 |
| *KIF18A* | kinesin family member 18A | -0.043 | 0.866 | 0.012 |
| *ZBTB16* | zinc finger and BTB domain containing 16 | 0.040 | 0.104 | 0.116 |
| *NPM1P37* | nucleophosmin 1 pseudogene 37 | 0.038 | 0.006 | 0.194 |
| *TBC1D31* | TBC1 domain family member 31 | -0.037 | 0.117 | -0.112 |
| *FKRP* | fukutin related protein | -0.028 | 0.803 | -0.018 |
| *HEXDC* | hexosaminidase D | -0.028 | 0.168 | -0.098 |
| *ZNF222* | zinc finger protein 222 | -0.021 | 0.925 | 0.007 |
| *LRRC69* | leucine rich repeat containing 69 | 0.015 | 0.061 | 0.133 |
| *HMGCS1* | 3-hydroxy-3-methylglutaryl-CoA synthase 1 | 0.015 | 0.024 | 0.161 |
| *RP11-204C16.4* | YWHAZ pseudogene 10 | 0.013 | 0.018 | 0.168 |
| *SEC61A2* | SEC61 translocon subunit alpha 2 | -0.013 | 0.421 | 0.058 |
| *RPL34P27* | ribosomal protein L34 pseudogene 27 | -0.008 | 0.103 | -0.116 |
| *RP11-289I10.2* | EGFR-coamplified and overexpressed protein (ECOP) pseudogene | 0.002 | 0.046 | 0.142 |
| *SLC39A1* | solute carrier family 39 member 1 | 0.002 | 0.201 | 0.091 |
| *ANAPC15* | anaphase promoting complex subunit 15 | -0.002 | 0.038 | -0.148 |
|  | Associations with Antibody Avidity/AI |  |  |  |
| *STAU2* | staufen double-stranded RNA binding protein 2 | -0.156 | 0.002 | -0.216 |
| *C8orf59* | ribosomal biogenesis factor | -0.125 | 0.001 | -0.227 |
| *ZNF222* | zinc finger protein 222 | 0.113 | 0.0001 | 0.267 |
| *PUS7* | pseudouridine synthase 7 | -0.083 | 0.039 | -0.147 |
| *FKRP* | fukutin related protein | 0.078 | 0.001 | 0.233 |
| *C7orf31* | chromosome 7 open reading frame 31 | -0.067 | 0.064 | -0.132 |
| *MSRA* | methionine sulfoxide reductase A | -0.062 | 0.001 | -0.235 |
| *RP11-512F24.1* | heterogeneous nuclear ribonucleoprotein A3 (hnRNPA3) pseudogene | 0.055 | 0.008 | 0.188 |
| *RP11-204C16.4* | YWHAZ pseudogene 10 | -0.052 | 0.750 | 0.023 |
| *GPR75* | G protein-coupled receptor 75 | 0.048 | 0.002 | 0.219 |
| *TBC1D31* | TBC1 domain family member 31 | -0.046 | 0.021 | -0.164 |
| *SPSB1* | splA/ryanodine receptor domain and SOCS box containing 1 | 0.035 | 0.010 | 0.183 |
| *API5P1* | apoptosis inhibitor 5 pseudogene 1 | 0.031 | 0.007 | 0.190 |
| *UBR7* | ubiquitin protein ligase E3 component n-recognin 7 | 0.014 | 0.095 | 0.119 |
| *CNDP2* | carnosine dipeptidase 2 | -0.013 | 0.012 | -0.178 |
| *THOC3* | THO complex 3 | -0.011 | 0.026 | -0.159 |
| *IDH2* | isocitrate dehydrogenase (NADP(+)) 2 | -0.008 | 0.081 | -0.124 |
| *CRIPT* | CXXC repeat containing interactor of PDZ3 domain | 0.007 | 0.009 | 0.185 |
| *RNASE4* | ribonuclease A family member 4 | 0.007 | 0.456 | 0.053 |
| *GPR89A* | G protein-coupled receptor 89A | -0.006 | 0.110 | -0.114 |
| *SLCO4C1* | solute carrier organic anion transporter family member 4C1 | 0.006 | 0.049 | 0.140 |
| *FGFR4* | fibroblast growth factor receptor 4 | 0.003 | 0.052 | 0.138 |

* The analysis was performed as outlined in Statistical analysis. Identified genes are displayed along with their beta coefficient from the lasso model and the Spearman correlation with the immune outcome residuals. Of note, the Spearman correlation p-value is based on “per gene” analysis. Thus, the gene identified/selected by the joint analysis approach may be jointly significant, but individually may not be significant.

**Supplementary Table 4** Results from SCCA and lasso regression modeling results of Day 8 B cell transcriptomic markers associated with MV-specific humoral immune response following MMR vaccination

| **Gene Symbol** | **Description** | **Beta**  **Coefficient** | **Spear. p-value** | **Spearman**  **Correlation**  **Coefficient** |
| --- | --- | --- | --- | --- |
|  | **Associations with Neut. Ab miu/ml** |  |  |  |
| *GNB2* | G protein subunit beta 2 | -0.248 | 0.001 | -0.239 |
| *RWDD3* | RWD domain containing 3 | -0.155 | 0.001 | -0.240 |
| *PUS7* | pseudouridine synthase 7 | -0.154 | 0.031 | -0.153 |
| *YWHAG* | tyrosine 3-monooxygenase/tryptophan 5-monooxygenase activation protein gamma | -0.133 | 0.058 | 0.135 |
| *DCAF7* | DDB1 and CUL4 associated factor 7 | 0.125 | 0.026 | -0.158 |
| *ASAH2B* | N-acylsphingosine amidohydrolase 2B | 0.116 | 0.025 | 0.159 |
| *GPN1* | GPN-loop GTPase 1 | 0.116 | 0.001 | 0.236 |
| *ATP13A4* | ATPase 13A4 | -0.115 | 0.032 | -0.153 |
| *TDG* | thymine DNA glycosylase | 0.114 | 0.002 | 0.218 |
| *ZMYM6NB* | transmembrane protein 35B | 0.112 | 0.076 | -0.127 |
| *AL158801.1* | ubiquitin-conjugating enzyme E2L 3 (UBE2L3) pseudogene | 0.104 | 0.006 | 0.195 |
| *LYSMD4* | LysM domain containing 4 | 0.100 | 0.001 | 0.226 |
| *MTSS1L* | MTSS I-BAR domain containing 2 | 0.097 | 0.014 | 0.175 |
| *SLC38A6* | solute carrier family 38 member 6 | -0.096 | 0.055 | -0.137 |
| *FAIM* | Fas apoptotic inhibitory molecule | 0.090 | 0.002 | 0.222 |
| *IPPK* | inositol-pentakisphosphate 2-kinase | 0.090 | 0.011 | 0.181 |
| *C11orf68* | chromosome 11 open reading frame 68 | -0.090 | 0.012 | -0.177 |
| *FLVCR1* | FLVCR heme transporter 1 | 0.088 | 0.003 | 0.211 |
| *DCUN1D4* | defective in cullin neddylation 1 domain containing 4 | 0.088 | 0.0002 | 0.263 |
| *RP4-717I23.2* | ribosomal protein L39 (RPL39) pseudogene | -0.083 | 0.0001 | -0.278 |
| *ERCC6-PGBD3* | chimeric ERCC6-PGBD3 protein | -0.083 | 0.029 | 0.155 |
| *TMX2-CTNND1* | TMX2-CTNND1 readthrough/NMD candidate | -0.082 | 0.007 | -0.192 |
| *RP11-79D8.2* | pseudogene | 0.081 | 0.062 | 0.133 |
| *DSP* | desmoplakin | -0.080 | 0.002 | -0.216 |
| *IL16* | interleukin 16 | 0.076 | 0.373 | -0.064 |
| *FAM133DP* | family with sequence similarity 133 member D, pseudogene | 0.075 | 0.009 | 0.184 |
| *PTBP2* | polypyrimidine tract binding protein 2 | -0.074 | 0.094 | 0.119 |
| *PHGDH* | phosphoglycerate dehydrogenase | -0.070 | 0.169 | -0.098 |
| *HMGB3* | high mobility group box 3 | -0.067 | 0.116 | 0.112 |
| *RGS14* | regulator of G protein signaling 14 | 0.067 | 0.079 | -0.125 |
| *EXTL2* | exostosin like glycosyltransferase 2 | 0.066 | 0.003 | 0.208 |
| *SARM1* | sterile alpha and TIR motif containing 1 | -0.065 | 0.416 | -0.058 |
| *ACTG1P4* | actin gamma 1 pseudogene 4 | -0.063 | 0.184 | -0.095 |
| *SLC25A20* | solute carrier family 25 member 20 | -0.063 | 0.036 | -0.149 |
| *B4GALT4* | beta-1,4-galactosyltransferase 4 | -0.063 | 0.019 | 0.167 |
| *NT5DC3* | 5'-nucleotidase domain containing 3 | -0.059 | 0.097 | -0.118 |
| *PI16* | peptidase inhibitor 16 | -0.057 | 0.005 | -0.197 |
| *ZNF33B* | zinc finger protein 33B | -0.055 | 0.043 | -0.144 |
| *TMEM43* | transmembrane protein 43 | 0.053 | 0.213 | -0.089 |
| *TRIM3* | tripartite motif containing 3 | 0.053 | 0.001 | 0.226 |
| *POU3F1* | POU class 3 homeobox 1 | -0.052 | 0.210 | 0.090 |
| *BEX2* | brain expressed X-linked 2 | 0.051 | 0.002 | 0.217 |
| *FAM117A* | family with sequence similarity 117 member A | 0.051 | 0.048 | -0.141 |
| *ABCC10* | ATP binding cassette subfamily C member 10 | -0.046 | 0.008 | -0.189 |
| *CH17-3B23.1* | NBPF member 26 | 0.045 | 0.013 | 0.176 |
| *LYL1* | LYL1 basic helix-loop-helix family member | -0.044 | 0.013 | -0.175 |
| *QSER1* | glutamine and serine rich 1 | -0.042 | 0.018 | -0.168 |
| *AKAP3* | A-kinase anchoring protein 3 | -0.042 | 0.005 | -0.197 |
| *RP11-122G18.7* | ribosomal protein S2 (RPS2) pseudogene | 0.041 | 0.022 | 0.163 |
| *PRUNE* | prune exopolyphosphatase 1 | -0.040 | 0.001 | -0.237 |
| *CTD-3148I10.9* | novel protein | -0.040 | 0.011 | -0.180 |
| *TIMM8B* | translocase of inner mitochondrial membrane 8 homolog B | -0.040 | 0.043 | -0.144 |
| *KNOP1* | lysine rich nucleolar protein 1 | -0.040 | 0.003 | -0.207 |
| *ZNF215* | zinc finger protein 215 | 0.040 | 0.008 | 0.188 |
| *NMD3* | NMD3 ribosome export adaptor | 0.039 | 0.001 | 0.237 |
| *WDR5B* | WD repeat domain 5B | -0.038 | 0.001 | -0.227 |
| *RAB21* | RAB21, member RAS oncogene family | -0.036 | 0.129 | 0.108 |
| *SH3BP4* | SH3 domain binding protein 4 | -0.035 | 0.039 | -0.147 |
| *ZNF367* | zinc finger protein 367 | -0.034 | 0.035 | 0.150 |
| *GTF2F2* | general transcription factor IIF subunit 2 | -0.034 | 0.171 | 0.098 |
| *RASSF7* | Ras association domain family member 7 | -0.033 | 0.005 | -0.198 |
| *TSFM* | Ts translation elongation factor, mitochondrial | 0.030 | 0.028 | 0.156 |
| *CRTAP* | cartilage associated protein | -0.027 | 0.420 | -0.058 |
| *RNF222* | ring finger protein 222 | -0.027 | 0.065 | -0.131 |
| *PSMA2* | proteasome 20S subunit alpha 2 | 0.026 | 0.009 | 0.185 |
| *PITPNM2* | phosphatidylinositol transfer protein membrane associated 2 | -0.026 | 0.036 | -0.149 |
| *GPD2* | glycerol-3-phosphate dehydrogenase 2 | 0.024 | 0.001 | 0.226 |
| *RP11-488C13.1* | ribosomal protein L22 pseudogene 2 | 0.023 | 0.012 | 0.179 |
| *PIGBOS1* | PIGB opposite strand 1 | 0.022 | 0.128 | -0.108 |
| *KBTBD8* | kelch repeat and BTB domain containing 8 | -0.022 | 0.851 | 0.013 |
| *NAT1* | N-acetyltransferase 1 | -0.018 | 0.126 | 0.109 |
| *CYB5D2* | cytochrome b5 domain containing 2 | -0.018 | 0.006 | -0.196 |
| *CNKSR2* | connector enhancer of kinase suppressor of Ras 2 | 0.015 | 0.132 | 0.107 |
| *RALBP1* | ralA binding protein 1 | 0.014 | 0.051 | -0.139 |
| *RAB28* | RAB28, member RAS oncogene family | -0.011 | 0.048 | -0.141 |
| *VPREB3* | V-set pre-B cell surrogate light chain 3 | 0.010 | 0.008 | -0.189 |
| *CCDC81* | coiled-coil domain containing 81 | 0.010 | 0.080 | 0.125 |
| *C17orf62* | cytochrome b-245 chaperone 1 | 0.009 | 0.020 | -0.165 |
| *CDK2AP1* | cyclin dependent kinase 2 associated protein 1 | 0.009 | 0.006 | 0.194 |
| *LGALSL* | galectin like | 0.008 | 0.004 | 0.204 |
| *MRPL11* | mitochondrial ribosomal protein L11 | 0.008 | 0.014 | 0.174 |
| *DDX52* | DExD-box helicase 52 | 0.008 | 0.0001 | 0.267 |
| *NDUFAB1* | NADH:ubiquinone oxidoreductase subunit AB1 | -0.007 | 0.039 | -0.147 |
| *INO80D* | INO80 complex subunit D | -0.004 | 0.151 | 0.103 |
| *ATP6V1E2* | ATPase H+ transporting V1 subunit E2 | -0.004 | 0.014 | -0.174 |
| *IMPA1* | inositol monophosphatase 1 | 0.003 | 0.005 | 0.199 |
| *NXPE3* | neurexophilin and PC-esterase domain family member 3 | -0.003 | 0.042 | -0.145 |
| *RNF138* | ring finger protein 138 | -0.003 | 0.037 | 0.148 |
| *HIVEP2* | HIVEP zinc finger 2 | 0.003 | 0.016 | -0.171 |
| *LY75-CD302* | LY75-CD302 readthrough | 0.002 | 0.054 | -0.137 |
| *THOC3* | THO complex 3 | -0.002 | 0.003 | -0.207 |
| *TRIM59* | tripartite motif containing 59 | 0.002 | 0.013 | 0.177 |
| *ACAT2* | acetyl-CoA acetyltransferase 2 | 0.001 | 0.035 | 0.150 |
| *EVI5* | ecotropic viral integration site 5 | 0.0005 | 0.009 | 0.186 |
|  | Associations with ELISA binding Ab/SI |  |  |  |
| *AARS* | alanyl-tRNA synthetase 1 | 0.182 | 0.700 | -0.028 |
| *EXTL2* | exostosin like glycosyltransferase 2 | 0.156 | 0.002 | 0.220 |
| *ABHD17C* | abhydrolase domain containing 17C, depalmitoylase | 0.134 | 0.004 | 0.204 |
| *LMTK2* | lemur tyrosine kinase 2 | -0.124 | 0.652 | -0.032 |
| *CAPN10* | calpain 10 | -0.124 | 0.018 | -0.168 |
| *ATP6V1E2* | ATPase H+ transporting V1 subunit E2 | -0.124 | 0.002 | -0.221 |
| *PHGDH* | phosphoglycerate dehydrogenase | -0.100 | 0.032 | -0.153 |
| *RFK* | riboflavin kinase | 0.100 | 0.016 | 0.172 |
| *WDR5B* | WD repeat domain 5B | -0.095 | 0.002 | -0.220 |
| *CRTAP* | cartilage associated protein | -0.094 | 0.154 | -0.102 |
| *CDC26* | cell division cycle 26 | -0.080 | 0.080 | -0.125 |
| *NDUFAB1* | NADH:ubiquinone oxidoreductase subunit AB1 | -0.079 | 0.061 | -0.133 |
| *TIMM23* | translocase of inner mitochondrial membrane 23 | -0.073 | 0.971 | 0.003 |
| *CR1* | complement C3b/C4b receptor 1 (Knops blood group) | -0.073 | 0.021 | -0.163 |
| *TAF4B* | TATA-box binding protein associated factor 4b | 0.072 | 0.002 | 0.222 |
| *RP5-1021I20.4* | novel protein | -0.069 | 0.776 | 0.020 |
| *DSP* | desmoplakin | -0.067 | 0.179 | -0.096 |
| *C1orf123* | CXXC motif containing zinc binding protein | -0.065 | 0.043 | -0.144 |
| *SYNGAP1* | synaptic Ras GTPase activating protein 1 | 0.065 | 0.784 | 0.020 |
| *ZNF267* | zinc finger protein 267 | -0.063 | 0.306 | 0.073 |
| *HYI* | hydroxypyruvate isomerase (putative) | 0.063 | 0.069 | 0.129 |
| *C9orf64* | chromosome 9 open reading frame 64 | -0.062 | 0.113 | -0.113 |
| *MARK2* | microtubule affinity regulating kinase 2 | 0.062 | 0.905 | -0.009 |
| *C8orf59* | ribosomal biogenesis factor | -0.060 | 0.010 | -0.182 |
| *UBE2B* | ubiquitin conjugating enzyme E2 B | -0.058 | 0.633 | 0.034 |
| *TDG* | thymine DNA glycosylase | 0.057 | 0.056 | 0.136 |
| *PI16* | peptidase inhibitor 16 | -0.056 | 0.063 | -0.132 |
| *ACTG1P4* | actin gamma 1 pseudogene 4 | -0.055 | 0.046 | -0.142 |
| *MSMO1* | methylsterol monooxygenase 1 | 0.055 | 0.114 | 0.113 |
| *RP4-717I23.2* | ribosomal protein L39 (RPL39) pseudogene | -0.050 | 0.023 | -0.162 |
| *MRPL11* | mitochondrial ribosomal protein L11 | 0.046 | 0.114 | 0.113 |
| *RP4-559A3.7* | novel protein | -0.043 | 0.005 | -0.200 |
| *SARM1* | sterile alpha and TIR motif containing 1 | -0.041 | 0.049 | -0.140 |
| *NT5DC3* | 5'-nucleotidase domain containing 3 | -0.041 | 0.389 | -0.062 |
| *UFD1L* | ubiquitin recognition factor in ER associated degradation 1 | 0.040 | 0.132 | 0.108 |
| *BTG3* | BTG anti-proliferation factor 3 | 0.038 | 0.040 | 0.146 |
| *ACAT2* | acetyl-CoA acetyltransferase 2 | 0.038 | 0.029 | 0.155 |
| *THOC3* | THO complex 3 | -0.038 | 0.094 | -0.119 |
| *SH3BP4* | SH3 domain binding protein 4 | -0.030 | 0.289 | -0.076 |
| *NEU3* | neuraminidase 3 | -0.028 | 0.010 | -0.183 |
| *COG8* | component of oligomeric golgi complex 8 | 0.026 | 0.581 | -0.039 |
| *LDHAP4* | lactate dehydrogenase A pseudogene 4 | 0.023 | 0.088 | 0.122 |
| *KDM8* | lysine demethylase 8 | -0.021 | 0.104 | -0.116 |
| *PITPNM2* | phosphatidylinositol transfer protein membrane associated 2 | -0.021 | 0.110 | -0.114 |
| *C1GALT1C1* | C1GALT1 specific chaperone 1 | -0.020 | 0.056 | -0.136 |
| *TMEM256-PLSCR3* | phospholipid scramblase 3 | -0.020 | 0.163 | -0.100 |
| *ATP13A4* | ATPase 13A4 | -0.016 | 0.087 | -0.122 |
| *TMEM123* | transmembrane protein 123 | 0.013 | 0.117 | 0.112 |
| *TRIM3* | tripartite motif containing 3 | 0.012 | 0.032 | 0.152 |
| *CCSAP* | centriole, cilia and spindle associated protein | 0.011 | 0.061 | 0.133 |
| *C11orf68* | chromosome 11 open reading frame 68 | -0.010 | 0.146 | -0.104 |
| *BEX2* | brain expressed X-linked 2 | 0.010 | 0.027 | 0.157 |
| *RABL3* | RAB, member of RAS oncogene family like 3 | 0.010 | 0.296 | 0.075 |
| *RP11-632C17__A.1* | ribosomal protein L29 (RPL29) pseudogene | -0.009 | 0.056 | -0.136 |
| *INO80D* | INO80 complex subunit D | 0.009 | 0.069 | 0.130 |
| *YAF2* | YY1 associated factor 2 | 0.008 | 0.056 | 0.136 |
| *RP11-488C13.1* | ribosomal protein L22 pseudogene 2 | 0.008 | 0.050 | 0.140 |
| *ZNF23* | zinc finger protein 23 | 0.007 | 0.086 | 0.122 |
| *C22orf46* | chromosome 22 putative open reading frame 46 | -0.005 | 0.085 | -0.123 |
| *CH17-3B23.1* | NBPF member 26 | 0.004 | 0.153 | 0.102 |
| *WDR81* | WD repeat domain 81 | -0.004 | 0.033 | -0.152 |
| *JOSD2* | Josephin domain containing 2 | -0.004 | 0.053 | -0.138 |
| *TIMM8B* | translocase of inner mitochondrial membrane 8 homolog B | -0.004 | 0.291 | -0.075 |
| *CCDC64* | BICD family like cargo adaptor 1 | -0.004 | 0.077 | -0.126 |
| *TMEM186* | transmembrane protein 186 | -0.002 | 0.001 | -0.239 |
| *DENND2D* | DENN domain containing 2D | 0.001 | 0.620 | 0.035 |

* The analysis was performed as outlined in Statistical analysis. Identified genes are displayed along with their beta coefficient from the lasso model and the Spearman correlation with the immune outcome residuals. Of note, the Spearman correlation p-value is based on “per gene” analysis. Thus, the gene identified/selected by the joint analysis approach may be jointly significant, but individually may not be significant.

**Supplementary Fig.1**  Immune response summary of the study subjects


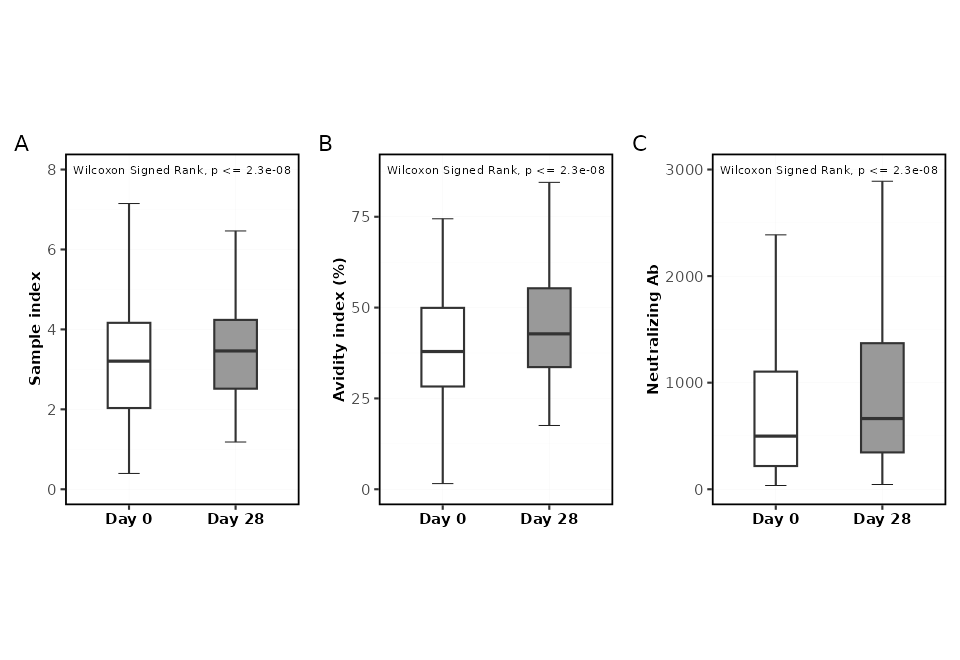


Box plots summarizing: A. Day 0 and Day 28 binding antibody sample index/SI; B. Day 0 and Day 28 antibody avidity index; and C. Day 0 and Day 28 Neutralizing Ab. The line indicates the median of the immune response measure in our cohort, while the whiskers indicate 25% and 75% IQR. The p-values (Wilcoxon signed rank test) demostrate the significant upregulation of Day 28 immune outcomes (post MMR3) compared to baseline (Day 0) immune outcomes.

**Supplementary Fig.2**  Heatmap of Day 0 gene expression patterns


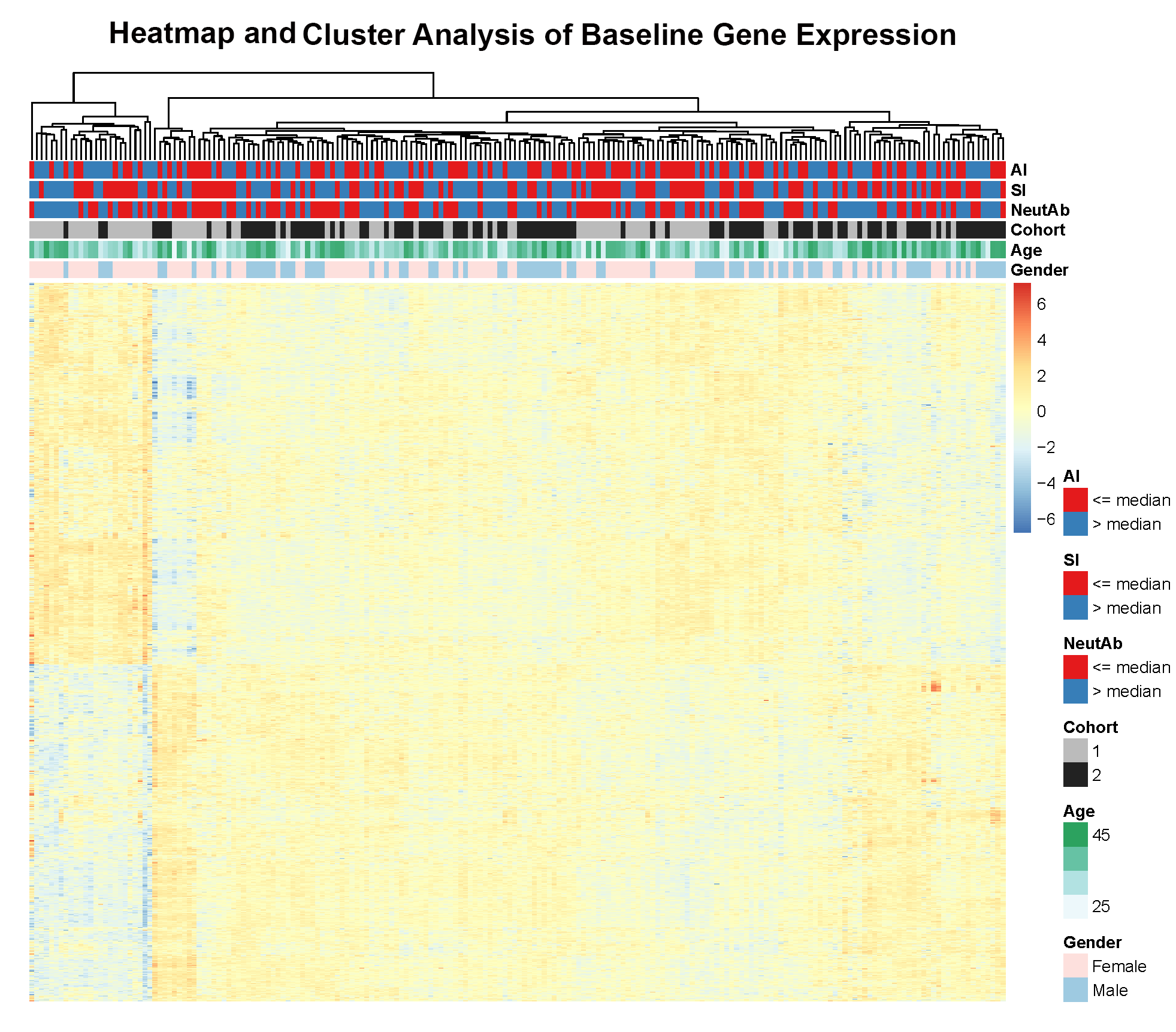


Heatmap of Day 0 (baseline) gene expression patterns of the significant genes (FDR < 0.1) from “per gene” analysis across covariates (sex, age, subcohort) and MV-specific immune response outcomes (Day 28 – Day 0 difference): Neutralizing Ab (Naut.Ab),SI (Sample Index/Binding Ab) and AI (Avidity Index).

**Supplementary Fig.3**  Heatmap of Day 8 gene expression patterns


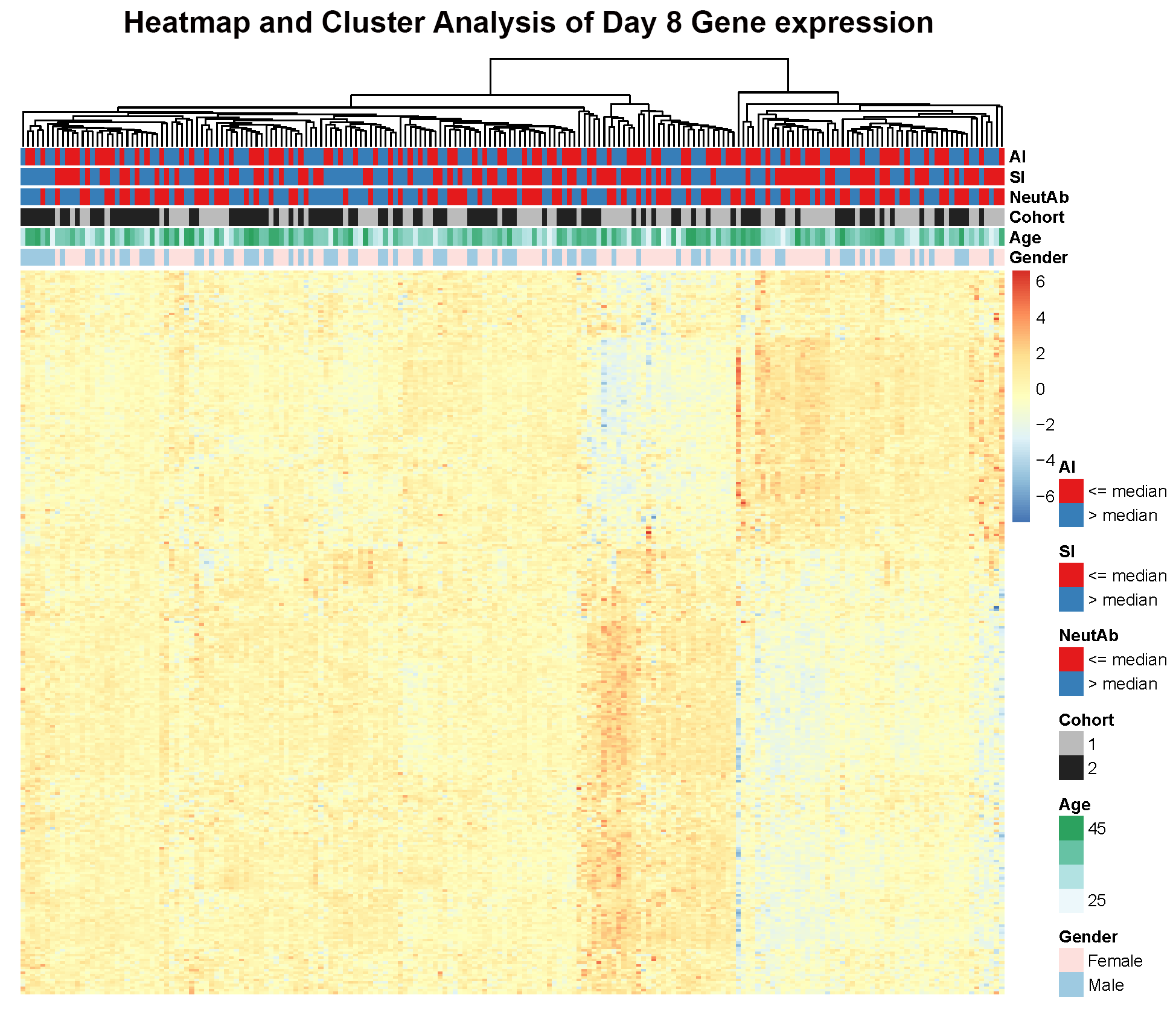


Heatmap of Day 8 gene expression patterns of the significant genes (FDR < 0.1) from “per gene” analysis across covariates (sex, age, subcohort) and MV-specific immune response outcomes (Day 28 – Day 0 difference): Neutralizing Ab (Naut.Ab),SI (Sample Index/Binding Ab) and AI (Avidity Index).
